# Size Estimation of Grasped Objects Using a Soft Pneumatic Gripper Integrated with a Piezoresistive CNT/PDMS Sensor

**DOI:** 10.3390/mi17060668

**Published:** 2026-05-28

**Authors:** Wongi Hong, Jaehoon Jeong, Kun-Woo Nam, Won-Jin Kim, Youngjae Cho, Eojin Ji, Taehyun Park, Dong Hun Lee, Sung-Hoon Park

**Affiliations:** Department of Mechanical Engineering, Soongsil University, 369 Sangdo-ro, Dongjak-Gu, Seoul 06978, Republic of Korea; ghddnjsrl0844@gmail.com (W.H.); wogns1218@soongsil.ac.kr (J.J.); kwn1522@naver.com (K.-W.N.); dnjswls0214@gmail.com (W.-J.K.); forcky9@soongsil.ac.kr (Y.C.); wldjwls314@gmail.com (E.J.); taehyunpark@ssu.ac.kr (T.P.)

**Keywords:** carbon nanotube, pressure sensor, piezoresistive effect, soft gripper

## Abstract

Soft pneumatic grippers are well-suited for grasping irregular objects owing to their inherent compliance and ability to adapt to a wide range of shapes and sizes. However, their ability to quantitatively estimate object size during the grasping process remains limited. To address this limitation, this study proposes a soft pneumatic gripper integrated with a piezoresistive CNT/PDMS composite sensor and investigates the feasibility of object size estimation using only sensor signals. The pressure-sensing characteristics of the CNT/PDMS sensor were evaluated over a pressure range of 0–500 kPa, and the 1 wt% CNT/PDMS sensor exhibited the highest sensitivity of approximately 0.016 kPa^−1^ in the initial linear pressure region. To this end, the normalized resistance response under applied pneumatic pressure was analyzed independent of external visual information, and a size estimation method was established based on the relationship between initial contact pressure and object diameter. Grasping experiments using spherical objects of varying diameters revealed that the resistance response patterns were clearly distinguishable according to object size, with larger objects exhibiting significant resistance changes at lower applied pressures. These findings demonstrate the feasibility of estimating the size of a grasped object based on the grasp onset pressure derived from the sensor response. The results of this study provide a foundation for future soft robotic systems capable of recognizing contact conditions and object size through sensor-based feedback. Furthermore, these findings may be extended to adaptive manipulation technologies involving real-time pneumatic pressure control.

## 1. Introduction

Elastomer-based pneumatic soft grippers exhibit greater compliance and deformability than conventional rigid grippers [1,2,3,4,5], allowing them to grasp irregularly shaped or fragile objects more safely. Owing to these advantages, soft pneumatic grippers have attracted considerable attention across diverse application fields, including agriculture [6,7], biomedicine [8], and industrial automation [9]. However, in practical applications, the ability to simply grasp an object is insufficient. The gripper must also be capable of recognizing the contact state with the object in real time and being controlled accordingly during the grasping process [10,11]. In particular, object size information plays an important role in determining an appropriate pneumatic pressure level and in preventing object damage caused by excessive compression or slippage caused by insufficient gripping force. To acquire such contact and size information, sensor technologies that can be directly integrated into the grasping process are required. In addition, given the soft grippers undergo large deformation during operation, the sensors must also possess sufficient flexibility to accommodate such deformation. However, widely used commercial sensors generally exhibit high mechanical stiffness, making them difficult to attach to curved surfaces and limiting their ability to effectively accommodate the deformation of soft grippers [12]. Therefore, flexible sensors for soft pneumatic grippers should not only provide structural flexibility and conformability to curved surfaces, but also enable stable detection of contact information generated during grasping.

In previous studies on flexible sensors integrated with soft grippers, bending or curvature sensors have been widely used to measure gripper deformation and to estimate grasping state or object size based on the resulting signals [13,14,15]. Such approaches are useful because they can reflect the shape change and overall behavior of soft actuators. In contrast, the present study focuses not on the global deformation of the actuator itself, but on the onset of actual contact with the grasped object and the corresponding response during the contact process. Accordingly, a pressure sensor was employed because it can more directly reflect the local interactions occurring at the contact interface. Since a pressure sensor captures the response in the region where the object and gripper come into contact, it enables a relatively intuitive interpretation of the initial contact point and the contact state, making it suitable for size estimation based on the response change during pneumatic actuation. Flexible pressure sensors can be classified according to their sensing mechanisms into piezoelectric [16,17], triboelectric [18,19], capacitive [20,21], and piezoresistive [22,23] types, each of which exhibits distinct operating characteristics. Piezoelectric sensors offer high sensitivity and excellent linearity, but they have limitations in measuring static pressure [24]. Triboelectric sensors can employ a wide variety of materials, although their sensing performance may be affected by changes in external environmental conditions [25]. Capacitive sensors provide high sensitivity and low power consumption, but they are susceptible to noise caused by external interference [26]. Among these sensing mechanisms, piezoresistive sensors have been widely employed in flexible pressure-sensing applications because of their simple device architecture, straightforward resistance-based signal readout, and relatively facile fabrication process [27,28,29,30].

In addition, they are well suited for composite-based designs and can be readily integrated with flexible substrates and elastic structures, while their electrical responses to pressure changes can be interpreted relatively intuitively. These characteristics are well aligned with the objective of this study, which is to detect local interactions at the contact region during grasping and, based on these responses, estimate the onset of grasping and the size of the object. Therefore, a piezoresistive flexible pressure sensor was employed in this study.

Carbon-based nanomaterials have been widely utilized in the field of flexible sensors owing to their high electrical conductivity, chemical stability, and excellent mechanical properties [31,32]. Among them, carbon nanotubes (CNTs) are particularly suitable as sensing materials for piezoresistive sensors because of their high aspect ratio and favorable electrical characteristics, which enable the formation of effective conductive networks even at low filler contents. In addition, CNTs are advantageous for flexible electronic devices and sensor applications because they can maintain stable electrical responses under repeated mechanical deformation. Meanwhile, polydimethylsiloxane (PDMS) is a representative elastomer with high flexibility and elasticity, offering excellent mechanical compatibility with soft robotic structures [33]. CNT/PDMS composites, in which CNTs are dispersed within a PDMS matrix, can simultaneously provide mechanical flexibility and electrical sensitivity, and can exhibit piezoresistive responses based on changes in the conductive network under applied pressure [34]. These characteristics suggest that CNT/PDMS composites are suitable materials for flexible pressure sensors, particularly in terms of contact pressure sensing and structural integration with soft grippers.

To more clearly position the present work within the broader field of flexible pressure sensing, representative piezoresistive and capacitive pressure sensors reported in previous studies were compared in terms of sensing material or structure, pressure range, sensitivity, gauge factor where available, and primary function or application, as summarized in Table 1. Previous piezoresistive pressure sensors have demonstrated high sensitivity in low-pressure regimes through three-dimensional CNT conductive networks, wearable pressure sensing using MWCNT/PDMS composite structures, and broad-range pressure detection using MWCNT/PDMS-based architectures [35,36,37]. Capacitive pressure sensors have likewise achieved high low-pressure sensitivity or wide sensing ranges through microstructured electrodes, high-permittivity dielectric layers, and engineered interfacial structures [38,39]. However, these prior studies have primarily focused on pressure monitoring, wearable sensing, or tactile detection. In contrast, the present study integrates a CNT/PDMS piezoresistive pressure sensor directly into a soft pneumatic gripper and utilizes the resulting grasp onset pressure as a physically interpretable parameter for quantitative object-size estimation without relying on external vision-based systems.

**Figure 1:**
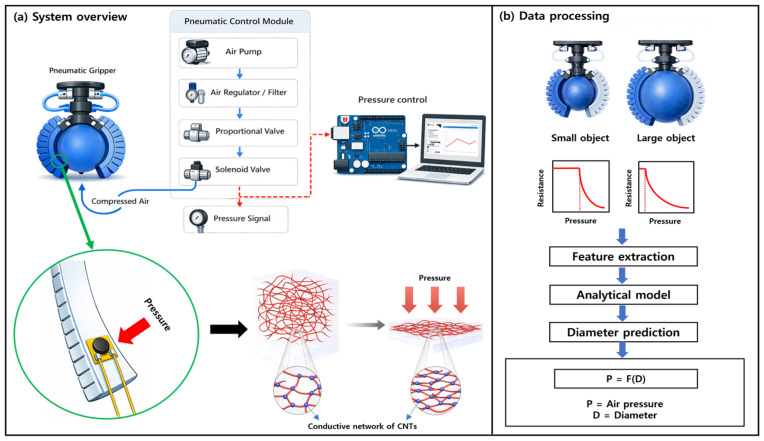
Scheme of the object size estimation system using a soft pneumatic gripper integrated with a CNT/PDMS-based flexible piezoresistive sensor: (**a**) Overall hardware configuration, including the pneumatic control module and data acquisition system, along with the piezoresistive sensing mechanism illustrating the change in the internal CNT conductive network under applied pressure; (**b**) Data processing procedure for deriving the final diameter prediction equation (P = F(D)) by applying feature extraction and an analytical model based on the varying resistance-pressure responses to objects of different diameters.

In this study, a piezoresistive flexible pressure sensor was fabricated by uniformly dispersing CNTs within a PDMS matrix through a 3-roll milling process, and the sensor was integrated into a pneumatic soft gripper. First, the electrical properties of the CNT/PDMS composite were evaluated by analyzing its dispersion characteristics and percolation behavior, and the pressure-sensing characteristics, sensitivity, and cyclic stability of the sensor were systematically investigated. In addition, grasping experiments were performed after attaching the fabricated sensor to the soft gripper, and the feasibility of estimating the size of the target object was examined by identifying the onset of grasping based on the normalized resistance change during the contact process. As a result, this study proposes a new approach for estimating object size using only a CNT/PDMS-based piezoresistive sensor directly integrated into a soft gripper, without relying on external vision-based systems. Figure 1 schematically illustrates the overall configuration of this study and the concept of object size estimation.

## 2. Materials and Methods

### 2.1. Materials

CNT and PDMS were used to fabricate the conductive composite for pressure sensing. The CNTs were obtained from KB-Element (Paju-si, Republic of Korea) and had an outer diameter of 5 nm, a bundle length of 10–20 μm, and a true density of 1.4 g/cm^3^. PDMS (Sylgard 184, Dow Corning, Midland, MI, USA) was used as the polymer matrix. In addition, polylactic acid (PLA) and an addition-cure silicone were used to fabricate the soft robotic gripper. PLA filament (PLA Basic, Bambu Lab, Shenzhen, China) was used for the interface components and actuator molds, and the soft actuators were fabricated using an addition-cure silicone elastomer (Smooth-Sil 950, Smooth-On, Macungie, PA, USA).

### 2.2. Fabrication of the CNT/PDMS-Based Pressure Sensor

**Figure 2:**
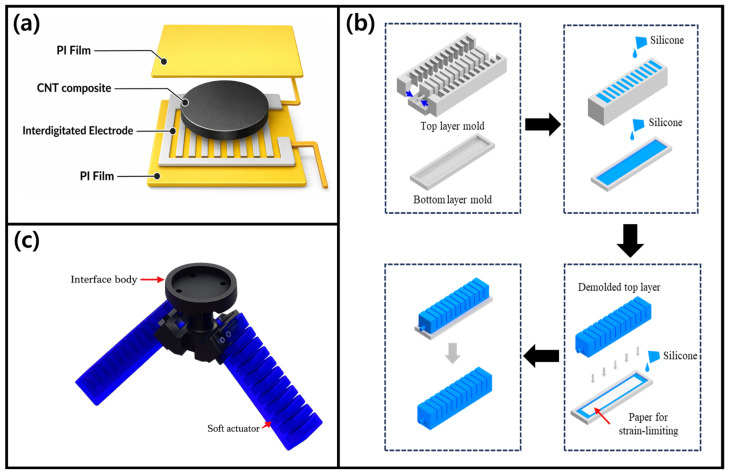
Fabrication and configuration of the sensor and soft gripper: (**a**) layer-by-layer structure and fabrication schematic of the CNT/PDMS piezoresistive sensor; (**b**) step-by-step fabrication process of the soft actuator, including mold preparation and silicone casting, bonding of each layer with insertion of a strain-limiting element, and demolding of the final integrated actuator; (**c**) overall configuration of the pneumatic soft gripper consisting of the interface body and soft actuators.

CNT/PDMS composites with various CNT contents were prepared to analyze the effect of conductive filler concentration on sensor characteristics. First, the PDMS base (Part A) and curing agent (Part B) were mixed at a mass ratio of 10:1, and CNTs were then added at predetermined contents. The mixture was stirred using a paste mixer (Daehwa Tech, Seoul, Republic of Korea) at 500 rpm for 30 s and 1500 rpm for 60 s, followed by additional dispersion for 5 min using a three-roll mill (Intech, Seongnam-si, Republic of Korea) [40]. After the dispersion process, the resulting composite was placed in a vacuum oven at room temperature for 20 min to remove any air bubbles trapped during the mechanical mixing and milling stages. Subsequently, the composite was hot-pressed using a hot press machine (Qmesys Inc., Uiwang-si, Republic of Korea) at 150 °C and 15 MPa for 1 h, producing a film with a thickness of 500 μm. The cured CNT/PDMS film was then cut into circular specimens with a diameter of 10 mm. Ag electrodes were deposited onto a flexible polyimide (PI) substrate using a metal sputter ion beam sputtering system (KVS-2000L, Gimpo-si, Republic of Korea). To maximize interfacial contact with the sensing layer within a limited device area, the electrodes were designed in an interdigitated electrode (IDE) structure, which provides an extended effective electrode length compared with conventional planar electrodes [41,42]. Moreover, the planar geometry of the IDE is well suited for implementation on flexible substrates and offers advantages in device integration through adjustment of the finger width and spacing. The fabricated IDE had an overall size of 10 mm × 10 mm, with a finger width of 0.30 mm and a gap of 0.50 mm between adjacent fingers. The sputtering process was conducted at room temperature under an RF power of 100 W, an Ar flow rate of 30 sccm, and a chamber pressure of 5 mTorr for 2 min 30 s. After electrode deposition, copper wires were attached using silver paste and cured at 100 °C for 1 h to establish stable electrical connections. The prepared CNT/PDMS composite film was then laminated onto the electrode layer, and the entire structure was encapsulated with a PI film to enhance mechanical stability, resulting in the final pressure sensor. Figure 2a shows a schematic representation of the fabricated pressure sensor, illustrating its multilayer structural configuration.

### 2.3. Fabrication of the Soft Pneumatic Gripper

The soft actuator was fabricated based on the pneumatic network (PneuNet) structure proposed by Mosadegh et al. [43]. Figure 2b schematically illustrates the fabrication process of the soft actuator. To define the internal air chamber geometry and the flat bottom base geometry of the actuator, 3D-printed molds were designed, and liquid silicone elastomer was poured into the molds to form each layer. The demolded top layer was then aligned and bonded onto the bottom layer, and a paper sheet was inserted between the two layers as a strain-limiting element to induce pneumatic expansion into bending motion. Finally, the assembled structure was fully cured after removal from the mold, resulting in an integrated soft actuator.

As shown in Figure 2c, the proposed pneumatic soft gripper was designed as a two-finger claw structure in which two soft actuators open outward. This configuration was intended to accommodate objects of various sizes, and the workspace was set to enable stable grasping of spherical objects with diameters ranging from 100 to 150 mm. The interface body at the top of the gripper was designed to be directly coupled to the UR5e end-effector of Universal Robots, and the soft actuators were fastened using insert nuts, bolts, and a separate fixing component. This mechanical fastening method provided stable support against the loads generated during pneumatic actuation and also allowed easy replacement of the actuators when necessary.

### 2.4. Gripper Actuation and Control System

The pneumatic circuit for the operation of the soft gripper is illustrated in the schematic in Figure 1. The pneumatic circuit begins as compressed air generated by the air pump is stored in the air tank, which reduces pressure pulsations and enables a stable supply. The stored air passes through a regulator and filter to be adjusted to constant pressure before being delivered to the proportional valve where the flow intensity is precisely modulated. The solenoid valve positioned after the proportional valve switches between the positive pressure line of the air pump and the negative pressure line of the vacuum pump to determine the inflation and deflation directions of the actuators. Finally, the compressed air passes through a pressure sensor and is supplied to the two soft actuators, which are connected via a single channel and operate simultaneously under the same pressure conditions.

The operation of this pneumatic circuit is integrated and controlled by an Arduino Mega (Arduino, Strambino, Italy). The solenoid valve is controlled in an on-off manner via a relay connected to the Arduino Mega to select the pressure source, while the proportional valve finely adjusts the airflow rate using a MOSFET–IRLZ44N (International Rectifier, El Segundo, CA, USA). The control system utilizes real-time pneumatic pressure data within the actuators obtained from the pressure sensor as feedback and performs a PID control algorithm to reach the set target pressure, thereby implementing stable grasping performance of the gripper.

### 2.5. Characterization and Test Conditions

To examine the dispersion state of CNTs in the fabricated composite, the specimens were fractured in liquid nitrogen, and the cross-sectional morphology and dispersion state were observed using a scanning electron microscope (SEM; Gemini SEM 300, ZEISS Inc., Baden-Württemberg, Germany). SEM observation was performed at an accelerating voltage of 1 kV.

CNT/PDMS composites with various CNT concentrations were prepared to investigate the electrical properties and percolation threshold as a function of conductive filler content. For each composition, five specimens with dimensions of 5 mm × 5 mm × 0.5 mm were fabricated. To improve the interfacial contact between the specimens and the electrodes, surface treatment was carried out for 300 s using a UV-ozone cleaner (JSE Co., Seoul, Republic of Korea). Silver paste (Protavic, Levallois-Perret, France) was then applied to both ends of each specimen to form electrodes, followed by curing at 150 °C for 1 h. The electrical resistance was measured using a four-point probe method with a digital multimeter (DMM 7510, Keithley, Cleveland, OH, USA) to minimize the effect of contact resistance. The measured values from the five specimens were averaged to evaluate the electrical properties of each composition.

The pressure-sensing characteristics of the composites were evaluated using a custom press system equipped with a strain gauge (NAMIL, Incheon, Republic of Korea). While pressure was applied to the specimens, changes in resistance were measured simultaneously using an LCR meter, and the compression test was conducted over a pressure range of 0–500 kPa at a rate of 1 mm/min. In addition, to evaluate response stability under cyclic loading, repeated loading-unloading tests were carried out for 100 cycles over a pressure range of 0–200 kPa for 2500 s.

The 1 wt% CNT/PDMS composite pressure sensor was integrated onto the inner surface of a pneumatic soft gripper for grasping experiments. Spherical specimens with diameters ranging from 100 to 150 mm, fabricated using a 3D printer (Bambu Lab, Shenzhen, China), were used as the grasping targets. The sensor resistance was monitored in real time while the applied pneumatic pressure was gradually increased from 0 to 60 kPa. Resistance measurements were performed using an LCR-6300 meter (Good Will Instrument Co., Taipei, Taiwan). Based on the correlation between the measured sensor response and the object diameter, a diameter prediction equation was established. The proposed equation was then validated using spherical specimens with arbitrary diameters within the measurement range, as well as actual fruits, in order to evaluate its validity and practical applicability to real grasping objects.

## 3. Results and Discussion

### 3.1. Morphology Analysis

**Figure 3:**
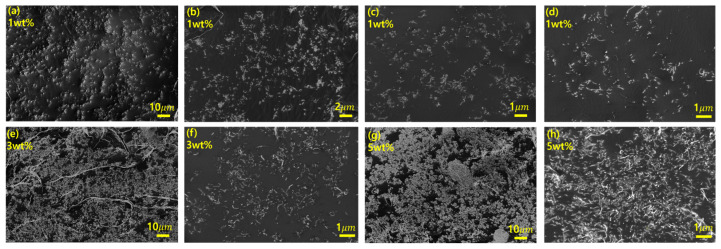
SEM images of CNT/PDMS composites with different CNT contents: (**a**–**d**) cross-sectional images of the 1 wt% CNT/PDMS composite observed at progressively increasing magnifications; (**e**) low-magnification image of the 3 wt% CNT/PDMS composite; (**f**) high-magnification image of the 3 wt% CNT/PDMS composite; (**g**) low-magnification image of the 5 wt% CNT/PDMS composite; and (**h**) high-magnification image of the 5 wt% CNT/PDMS composite.

Figure 3 presents SEM images of the fractured surfaces of CNT/PDMS composites with different CNT contents. Figure 3a–d shows cross-sectional SEM images of the 1 wt% CNT/PDMS composite observed at progressively increasing magnifications, providing a more detailed view of the CNT dispersion within the PDMS matrix. Figure 3e,f presents low- and high-magnification images of the 3 wt% CNT/PDMS composite, respectively, whereas Figure 3g,h shows low- and high-magnification images of the 5 wt% CNT/PDMS composite, respectively. SEM observations confirmed that the CNTs were generally well dispersed throughout the PDMS matrix through the 3-roll milling process. As the CNT content increased, the distribution density of CNTs within the composite also increased, accompanied by a higher frequency of CNT–CNT contacts and contact points. These changes are considered to have promoted the formation of conductive networks, thereby increasing the number of conductive pathways within the composite. Meanwhile, no distinct large-scale agglomerates were observed even at higher CNT loadings, indicating that the present process was effective in ensuring stable CNT dispersion.

### 3.2. Electrical Conductivity and Percolation Threshold

**Figure 4:**
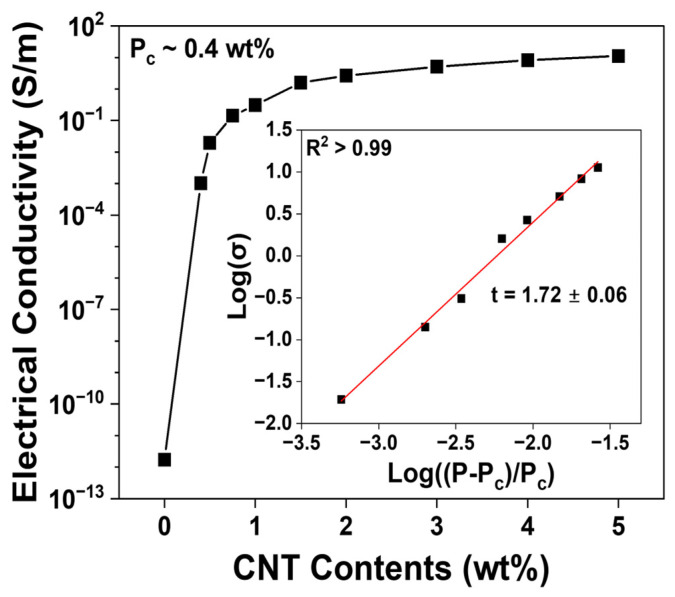
Electrical conductivity of CNT/PDMS composites as a function of CNT mass fraction (wt%). The electrical percolation threshold ($p_{c}$) was identified at approximately 0.4 wt%. Inset: log-log plot of electrical conductivity versus ($p-p_{c}$)/$p_{c}$ with the linear fit (red line) yielding a critical exponent (*t*) of 1.72 ± 0.06 and an *R^2^* value greater than 0.99.

To evaluate the electrical properties of the fabricated CNT/PDMS composites, the electrical conductivity and percolation threshold were investigated as a function of CNT content. Figure 4 presents the electrical conductivity of the composites as a function of CNT content (0.4, 0.5, 0.75, 1, 1.5, 2, 3, 4, and 5 wt%). The electrical conductivity was calculated from the measured resistance values using Equation (1):

(1) σ=L∕RA

where σ denotes the electrical conductivity, *R* is the electrical resistance, *A* is the cross-sectional area of the specimen, and *L* is the distance between the electrodes.

Owing to their high aspect ratio, CNTs readily form conductive networks within the polymer matrix, thereby enabling measurable electrical conductivity even at low filler contents. As the CNT content increased beyond a critical level, a typical percolation behavior was observed, characterized by an abrupt increase in conductivity by several orders of magnitude, which is attributed to the formation of continuous conductive pathways throughout the composite [44,45].

Such conductive behavior can be explained by both direct CNT-CNT contact and electron tunneling. In particular, electron tunneling enables charge transport across the thin PDMS insulating barrier between adjacent CNTs, thereby contributing to electrical conduction even in the absence of direct physical contact [46,47]. According to percolation theory, the relationship between composite conductivity and filler content can be expressed by Equation (2) [44,48]:

(2) $$\sigma_{c}=\sigma_{0}{\left(p-p_{c}\right)}^{t}$$

where $\sigma_{c}$ is the electrical conductivity of the composite, $\sigma_{0}$ is a proportionality constant, p is the CNT content, $p_{c}$ is the electrical percolation threshold, and *t* is the critical exponent. From Equation (2), the values of pc and t were estimated to be 0.4 wt% and 1.72 ± 0.06, respectively. These values are in good agreement with previously reported results, supporting the formation of a stable three-dimensional conductive network in the fabricated CNT/PDMS composites [49,50].

At CNT contents well above the percolation threshold (above 4 wt%), however, the increase in electrical conductivity became progressively less pronounced and eventually approached saturation. This behavior is attributed to the fact that a sufficiently dense conductive network had already been established within the matrix, such that additional CNT incorporation mainly increased the redundancy of the existing network rather than generating new conductive pathways.

### 3.3. Characterization of Piezoresistive Sensing Performance

To evaluate the piezoresistive characteristics of the fabricated CNT/PDMS sensors under applied pressure, pressure-loading experiments were conducted on sensors with different CNT contents. The experiments were performed using a custom-built pressing system, and the maximum applied pressure was set to 500 kPa considering the operating range of the load cell. The sensitivity was calculated from the initial linear region below 50 kPa using Equation (3) [51]:

(3) $$S=\left(\Delta R∕R_{0}\right)∕\Delta P$$

where S is the sensitivity, ΔR is the change in resistance, R_0_ is the initial resistance under no-pressure conditions, and ΔP is the change in applied pressure.

**Figure 5:**
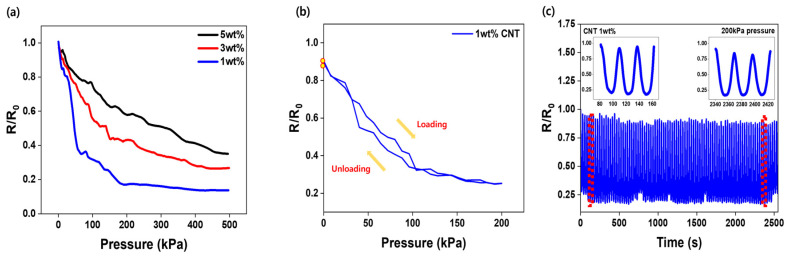
Pressure response, hysteresis behavior, and cyclic stability of the sensors with varying CNT content: (**a**) Normalized resistance change (R/R_0_) of 1, 3, and 5 wt% CNT/PDMS composites under applied pressure; (**b**) Normalized resistance response of the 1 wt% CNT/PDMS sensor during a loading–unloading pressure cycle up to 200 kPa, showing its hysteresis behavior. The yellow circles indicate the initial points of the loading and unloading curves. (**c**) Cyclic compression–unloading response of the 1 wt% CNT/PDMS sensor at 200 kPa for 100 cycles over 2500 s. The insets provide magnified views of the resistance response during the initial (~100 s) and final (~2400 s) stages, demonstrating the excellent repeatability and signal consistency of the sensor.

As shown in Figure 5a, all CNT/PDMS sensors exhibited a typical piezoresistive behavior in which R/R_0_ decreased with increasing pressure. In particular, the normalized resistance change in the 1 wt% sensor decreased to approximately 0.2 at 200 kPa, whereas the 3 wt% and 5 wt% sensors decreased to about 0.4 and 0.6, respectively, at the same pressure. This decrease in resistance can be attributed to two factors. First, the contact condition between the electrode and the composite was improved under applied pressure, resulting in reduced contact resistance [52]. Second, the distance between adjacent CNTs within the composite decreased under compression, which promoted the formation of additional conductive pathways [53,54]. In other words, the compressive deformation enhanced the connectivity of microscopic conductive networks and facilitated electron tunneling, leading to an overall reduction in electrical resistance.

Meanwhile, the difference in the magnitude of normalized resistance change depending on CNT content is associated with the initial formation state of the conductive network within the composite. Because CNTs are one-dimensional high-aspect-ratio fillers, effective conductive pathways can be formed even at relatively low concentrations. In particular, the 1 wt% CNT/PDMS composite is considered to possess a conductive network that is sufficiently formed to generate a measurable electrical response, while still remaining highly susceptible to pressure-induced rearrangement. Therefore, under compression, the reduction in inter-CNT distance and the formation of additional conductive pathways can produce a larger relative resistance change [50]. In contrast, as the CNT content increases, a sufficiently developed conductive network is already present, limiting the contribution of additional pathway formation under applied pressure. As a result, the resistance variation with pressure becomes smaller with increasing CNT content, and the sensitivity correspondingly decreases. A similar filler-content-dependent trend has been reported in previous CNT/PDMS piezoresistive pressure sensor studies, in which the 1 wt% composite exhibited higher pressure sensitivity than the 3 and 5 wt% composites [50]. Indeed, the sensitivities of the 1 wt%, 3 wt%, and 5 wt% sensors in the initial linear region were calculated to be 0.016 kPa^−1^, 0.009 kPa^−1^, and 0.005 kPa^−1^, respectively, indicating that the 1 wt% sensor exhibited the highest sensitivity.

In addition to pressure sensitivity, the gauge factor (GF) was calculated to further quantify the piezoresistive response of the CNT/PDMS sensors with respect to compressive deformation. The gauge factor is commonly defined as the relative resistance change per unit mechanical strain, as expressed in Equation (4) [55]:

(4) $$GF=\left(\Delta R∕R_{0}\right)∕\varepsilon$$

where GF is the gauge factor and ε is the compressive strain. In this study, ε was determined by dividing the measured compressive displacement by the initial thickness of the CNT/PDMS composite film.

Based on the experimentally measured compressive displacement and the corresponding normalized resistance change, the effective compressive GF values of the 1 wt%, 3 wt%, and 5 wt% sensors were calculated to be approximately −3.60, −1.57, and −1.51, respectively. The negative sign indicates that the electrical resistance decreased under compressive deformation. In terms of magnitude, the 1 wt% sensor exhibited the highest GF, consistent with its highest pressure sensitivity. Meanwhile, the 3 wt% and 5 wt% sensors showed comparable GF values. This tendency can be explained by the development of the conductive CNT network at higher filler contents. Once conductive pathways are sufficiently established, additional CNT incorporation does not necessarily produce a proportional increase in strain-induced resistance modulation, resulting in a reduced and less distinguishable GF response at higher CNT loadings [50,55].

Polymer-based pressure sensors may exhibit hysteresis due to their viscoelastic nature, in which the electrical response does not immediately return to its initial state after removal of the external load [56,57]. This behavior can lead to different response curves during loading and unloading, thereby reducing the stability and reliability of the sensor during repeated measurements. Therefore, the hysteresis behavior of the CNT/PDMS sensor was first evaluated using a single loading–unloading pressure sweep test with the 1 wt% CNT/PDMS sensor, which exhibited the highest pressure sensitivity. As shown in Figure 5b, the normalized resistance decreased during loading and increased again during unloading; however, the loading and unloading curves did not completely overlap, indicating the presence of measurable hysteresis. The hysteresis error was calculated using the following equation [58]:

(5) $$H=\frac{Y_{mn}-Y_{mp}}{Y_{max}-Y_{min}}\times 100$$

where Y_mn_ and Y_mp_ denote the sensor outputs on the unloading and loading branches, respectively, at the pressure point where the difference between the two branches is maximal, and Y_max_ and Y_min_ represent the maximum and minimum output values over the full loading–unloading response range. Based on this definition, the hysteresis error obtained from the single loading–unloading curve in Figure 5b was approximately 18.5%. This result indicates that the CNT/PDMS sensor exhibits a finite and non-negligible hysteretic response under pressure loading.

In addition to the single-cycle hysteresis evaluation, repeated loading–unloading tests were performed to examine the cyclic stability and repeatability of the sensor response. The test was conducted using the 1 wt% CNT/PDMS sensor by repeatedly applying and releasing a pressure of 200 kPa for 100 cycles over a total duration of 2500 s. When ten loading–unloading cycles were extracted from the repeated cyclic response and analyzed using the same hysteresis-error definition, the average hysteresis error was calculated to be 18.04%, which is comparable to the value obtained from the single loading–unloading pressure sweep. As shown in Figure 5c, the sensor exhibited a slight decrease in normalized resistance during the initial cycles, but maintained a relatively stable response during most of the subsequent cyclic loading conditions. The peak and baseline resistance values showed only limited variation after the initial period, suggesting that the sensor maintained reasonable cyclic repeatability under repeated compression.

The observed hysteresis and initial drift can be attributed to the viscoelastic deformation of the PDMS matrix and the microstructural rearrangement of the CNT conductive network during compression and release [59,60]. Under applied pressure, the distance between adjacent CNTs decreases, which may generate new conductive pathways or reduce the tunneling resistance between neighboring CNTs. During unloading, however, the polymer matrix and the conductive network may not immediately recover to their original configurations, resulting in a difference between the loading and unloading responses. In addition, repeated compression can induce gradual rearrangement, sliding, bending, or reorientation of CNTs within the PDMS matrix, leading to the initial drift observed during the early cycles. After repeated loading, the CNT network is considered to gradually approach a more stabilized conductive structure, which is consistent with the relatively stable response observed in the later cycles.

Although the CNT/PDMS sensor exhibited measurable hysteresis, its influence on the object-size estimation strategy proposed in this study is expected to be relatively limited. In the present approach, object size is not estimated from a bidirectional pressure–resistance relationship that requires both loading and unloading responses. Instead, the proposed method identifies the grasp onset pressure during a monotonic pressurization process, based on the initial sustained decrease in normalized resistance. Therefore, the unloading branch, which directly contributes to the hysteresis calculation, is not used in the regression model for object-size estimation. Nevertheless, hysteresis remains an important sensor characteristic when the device is used for repeated grasp–release operations or precise pressure monitoring under both loading and unloading conditions. The present results therefore provide a basis for understanding the hysteretic behavior of the CNT/PDMS sensor while clarifying that the proposed grasp-onset-based size estimation method mainly relies on the monotonic pressurization response.

### 3.4. Analysis of Sensor Response and Modeling of Size Correlation During Grasping

Before grasping experiments, the 1 wt% CNT/PDMS composite, which exhibited the highest sensitivity and cyclic stability in the previous experiments, was selected as the final pressure-sensing material. The selected pressure sensor was attached to the inner side surface of the soft pneumatic gripper at the region expected to directly contact the grasped object during actuator bending and was then applied to the grasping experiments.

The actuation pressure range of 0–60 kPa was selected based on the predefined operating range of the soft pneumatic gripper used in this study. This pressure regime was sufficient to produce progressive actuator deformation and stable grasping of the tested objects, and comparable low-pressure ranges have been adopted in previous studies on pneumatic soft grippers for grasping applications [61,62]. Therefore, the linear relationship between grasp onset pressure and object diameter presented here should be interpreted within the tested object sizes and the 0–60 kPa operating range of the present gripper system.

**Figure 6:**
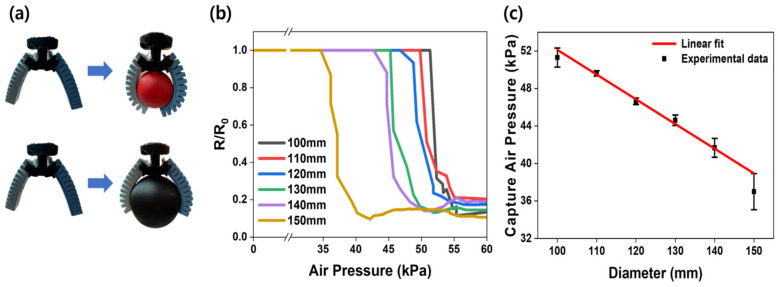
Grasping experiments and size estimation results using spherical specimens: (**a**) Snapshots of the soft pneumatic gripper grasping spherical specimens with different diameters; (**b**) Normalized resistance change (R/R_0_) as a function of applied air pressure during grasping; (**c**) Linear regression analysis showing the relationship between the grasping onset pressure (kPa) and the sphere diameter (mm).

As shown in Figure 6a, grasping experiments were conducted using spherical specimens with diameters ranging from 100 to 150 mm. Spherical specimens were selected to minimize geometric variables associated with object shape and to simplify the contact conditions during grasping, thereby enabling a more quantitative evaluation of the sensor response as a function of object diameter. During each experiment, the spherical specimen was carefully aligned and maintained at a predefined position so that contact occurred consistently at the same sensing region of the gripper. This procedure was adopted to minimize variations in grasp onset pressure that could arise from differences in the relative contact location between the object and the sensor. In this study, the onset of grasping was defined as the point at which the normalized resistance first decreased by more than 10% from its initial value, which was empirically determined as a threshold to reliably distinguish true contact from noise-induced fluctuations. This criterion was adopted to reduce false detection caused by noise and to more clearly identify the actual onset of contact and grasping.

Figure 6b shows the normalized resistance change in the sensor according to the applied pressure during grasping of the spherical specimens. The pneumatic pressure was increased from 0 to a maximum of 60 kPa, and the normalized resistance change decreased to approximately 0.1 when the specimen was maximally compressed. As the specimen diameter increased, the onset of grasping appeared at lower pressure levels, and the corresponding sensor response was also observed at an earlier stage. This indicates that contact and grasping between the gripper and the specimen begin at lower pressures for larger-diameter objects.

These observations suggest that the initial contact pressure varies depending on the specimen diameter. Accordingly, the relationship between specimen diameter and applied pneumatic pressure was analyzed. As the diameter increased, contact with the object began at lower pressures. Accordingly, a linear relationship was obtained, with the grasp onset pressure defined as the dependent variable (Y) and specimen diameter as the independent variable (X), indicating a consistent empirical correlation within the investigated diameter range. Figure 6c presents the relationship between grasp onset pressure and specimen diameter, together with the linear regression result and the standard deviation for each condition. The mean pressure and standard deviation at each data point were 51.31 ± 1.01 kPa for 100 mm, 49.62 ± 0.29 kPa for 110 mm, 46.65 ± 0.35 kPa for 120 mm, 44.62 ± 0.55 kPa for 130 mm, 41.67 ± 1.01 kPa for 140 mm, and 37.00 ± 1.91 kPa for 150 mm. Based on these data, the linear regression equation was derived as follows, where y represents the grasp onset pressure (kPa) and x represents the diameter of the grasped sphere (mm).

(6) y=−0.26376x+78.50392

The coefficient of determination was R^2^ = 0.98312, indicating a strong correlation between grasp onset pressure and specimen diameter. These results confirm that the diameter of the grasped object can be estimated by identifying the grasp onset pressure from the normalized resistance change in the sensor. Therefore, the proposed system can be utilized not only for simple contact detection but also for quantitative size estimation based on sensor-derived grasp onset behavior.

### 3.5. Reliability Assessment of the Size Estimation Model for Various Grasping Objects

Given that the previously derived linear regression equation was obtained from experiments using spherical specimens, additional validation was necessary to evaluate both the interpolation and extrapolation capability of the proposed model. Accordingly, two validation experiments were conducted to assess the validity of the derived equation.

**Figure 7:**
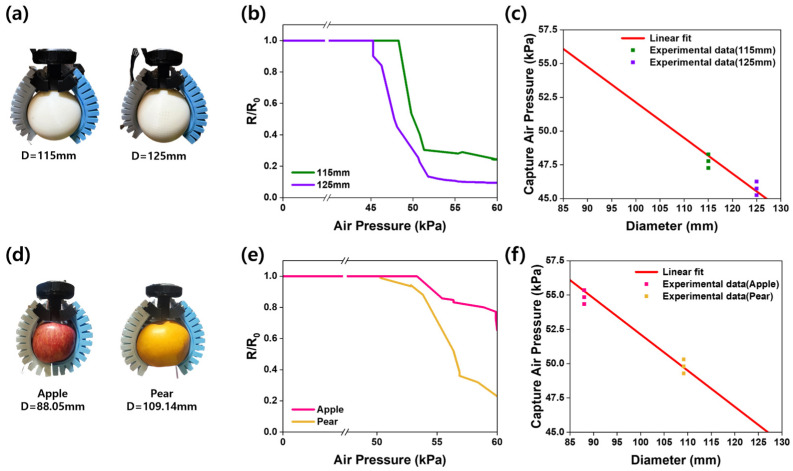
Validation results of the diameter estimation model: (**a**) Snapshots of the gripper grasping spherical specimens with arbitrary diameters (115 and 125 mm); (**b**) Normalized resistance change (R/R_0_) versus applied air pressure for the spherical specimens; (**c**) Comparison between the predicted values from the linear regression model and the experimental results for the spheres; (**d**) Snapshots of the gripper grasping real-world objects (apple and pear); (**e**) Normalized resistance change (R/R_0_) versus applied air pressure for the real fruits; (**f**) Validation of the estimation model’s applicability for predicting the size of unstructured real-world objects.

In the first validation experiment, two spherical specimens with arbitrary diameters within the measurement range were selected, and the experimental results were compared with the previously derived linear equation. Figure 7a shows the spherical specimens used in the grasping experiment, with diameters of 115 mm and 125 mm, respectively. Based on the regression equation, the predicted grasp onset pressures for these specimens were 48.17 kPa and 45.53 kPa, respectively. Figure 7b shows the normalized resistance change in the sensor as a function of the applied pneumatic pressure during grasping of the spherical specimens. Consistent with the previous experiments, grasping began at a lower pneumatic pressure for the larger-diameter sphere, and the corresponding resistance change was also observed at an earlier pressure range. In addition, the normalized resistance change decreased to approximately 0.1 at the maximum applied pressure. These results indicate that the correlation between grasp onset pressure and specimen diameter was maintained even in the intermediate diameter range.

Figure 7c presents the data points obtained from the validation experiment together with the previously derived linear regression line. The measured grasp onset pressures were 48.22, 47.25, and 47.27 kPa for the 115 mm specimen, and 46.26, 45.75, and 45.25 kPa for the 125 mm specimen, which showed overall good agreement with the regression equation. These findings demonstrate that the derived equation can be validly applied to spherical objects with arbitrary diameters within the measurement range.

In the second validation experiment, actual fruits with near-spherical shapes were grasped, and the results were compared with the derived linear regression equation. Figure 7d shows the fruits used in the grasping experiment. The diameters of the fruits were determined by measuring their maximum horizontal lengths using a vernier caliper, yielding values of 88.05 mm and 109.14 mm, respectively. Notably, the diameter of the apple was outside the range used in the previous spherical grasping experiments, thereby enabling evaluation of the extrapolation capability of the regression equation. Based on the derived linear equation, the predicted grasp onset pressures were 55.28 kPa for the apple and 49.72 kPa for the pear.

Figure 7e shows the normalized resistance change in the sensor as a function of the applied pneumatic pressure during fruit grasping. Similarly to the spherical specimen experiments, grasping of the larger fruit began at a lower pneumatic pressure, and the corresponding resistance change was observed at an earlier stage. As in the previous experiments, the pear exhibited a normalized resistance change approaching approximately 0.1 at the maximum applied pressure. In contrast, because grasping of the apple began at a relatively higher pressure, its resistance did not decrease substantially even at the maximum applied pressure. Figure 7f presents the data points obtained from the fruit grasping experiments together with the derived linear regression line.

For each fruit sample, grasping experiments were repeated 10 times. To concisely represent the experimentally observed range of grasp onset pressure while maintaining the clarity of the figure, the minimum, median, and maximum grasp onset pressure values obtained from the ten repeated trials are presented in Figure 7f. The corresponding values were 54.34, 54.84, and 55.35 kPa for the apple, and 49.29, 49.79, and 50.30 kPa for the pear, respectively. Because fruits do not have perfectly symmetric geometries unlike spherical specimens, relatively larger variations in sensor response were observed depending on the grasping position and shape. Nevertheless, the experimental data exhibited a trend consistent with that predicted by the linear equation, indicating that the proposed equation can be applied to the approximate size estimation of irregular objects with reasonable predictive reliability.

To further quantify the predictive performance of the derived linear regression model, the measured grasp onset pressures obtained from the validation experiments were converted into predicted object diameters using the inverse form of the regression equation. The predicted diameter, D_pred_, was calculated as follows:

(7) $$D_{pred}=\left(78.50392-P_{onset}\right)∕0.26376$$

where P_onset_ is the experimentally measured grasp onset pressure. Based on the mean grasp onset pressures obtained from the validation experiments, the predicted diameters were 117.17 mm for the 115 mm spherical specimen, 124.17 mm for the 125 mm spherical specimen, 89.70 mm for the apple, and 108.85 mm for the pear. These values showed good agreement with the corresponding actual diameters of 115.00, 125.00, 88.05, and 109.14 mm, respectively.

To quantitatively evaluate the prediction accuracy of the linear regression model, the mean absolute error (MAE), mean absolute percentage error (MAPE), and root mean square error (RMSE) were calculated, as these metrics are commonly used to assess prediction errors in continuous regression problems [63,64]. The corresponding equations are given as follows:

(8) $$MAE=\frac{1}{n}\sum_{i=1}^{n}\left|Di-{\hat{D}}_{i}\right|\;$$

(9) $$MAPE=\frac{100}{n}\sum_{i=1}^{n}\left|\frac{Di-\hat{D}i}{Di}\right|\;$$

(10) $$RMSE=\sqrt{\frac{1}{n}\sum_{i=1}^{n}{\left(Di-{\hat{D}}_{i}\right)}^{2}}$$

where Di is the actual diameter of the i-th validation object, ${\hat{D}}_{i}$ is the predicted diameter obtained from the regression equation, and n is the number of validation objects. Based on the four validation objects, the proposed linear regression model yielded an MAE of 1.24 mm, a MAPE of 1.17%, and an RMSE of 1.43 mm. These results quantitatively confirm that the proposed low-complexity linear regression model provides reasonably accurate size-estimation performance under the tested conditions.

## 4. Conclusions

In this study, a piezoresistive CNT/PDMS composite sensor was integrated into a soft pneumatic gripper to enable size estimation of grasped objects. The results demonstrated that the grasp onset pressure, defined from the normalized resistance response, exhibited a strong linear correlation with object diameter. This relationship enables size estimation using only internal sensor signals without the need for external vision-based systems. The proposed model was validated using both intermediate spherical specimens and real fruit samples with geometric variations, confirming its applicability within the investigated range and its potential applicability to practical objects. Despite the geometric variations in the real fruit samples, the sensor response showed consistent trends with the predicted behavior, indicating reliable estimation performance. Therefore, the proposed sensor-integrated system enables not only contact detection but also quantitative size estimation based on the grasp onset response, offering practical potential for real-time object classification and adaptive manipulation. This approach contributes to the development of intelligent soft robotic systems, particularly in applications such as automated harvesting and delicate object handling. However, because the quantitative relationship between grasp onset pressure and object size was primarily established using spherical objects under relatively well-defined contact conditions, the direct application of this relationship to irregularly shaped or non-spherical objects may require additional calibration or shape-dependent correction. In future work, the proposed approach will be further extended to objects with more diverse geometries by considering local contact conditions, object orientation, and surface features. Furthermore, machine learning-based prediction models will be explored in future work to address more complex and irregular object geometries, where the relationship between sensor responses and object dimensions may not be sufficiently captured by a simple linear regression model.

## Figures and Tables

**Table 1:** Comparison of representative flexible pressure sensors reported in previous studies and the present work in terms of sensing mechanism, structure, pressure range, sensitivity, gauge factor, and primary function.

Ref.	Sensing Mechanism	Sensing Material/Structure	Pressure Range	Sensitivity	Gauge Factor (GF)	Main Feature/Application
[35]	Piezoresistive	CNT-embedded 3D TPE conductive network	<0.2 kPa (high-sensitivity region)	136.8 kPa^−1^	6.85 under tensile strain	Highly sensitive low-pressure detection and human-motion monitoring
[36]	Piezoresistive	Spinous microstructured MWCNT/PDMS pressure sensor	0–31.83 kPa (high-sensitivity linear range)	0.026 kPa^−1^	Not reported	Wearable glove-integrated pressure sensing
[37]	Piezoresistive	MWCNT/PDMS composite pressure sensor	0–200 kPa; detectable up to 500 kPa	0.13% kPa^−1^	Not reported	Broad-range pressure sensing using a mold-printed MWCNT/PDMS conductive layer
[38]	Capacitive	Micro-array electrode and high-permittivity dielectric layer	0–2.5 kPa (low-pressure sensing range)	4.9 kPa^−1^	Not applicable	Highly sensitive low-pressure capacitive sensing for wearable monitoring
[39]	Capacitive	PDMS-based capacitive pressure sensor with engineered interfaces and a micropyramidal dielectric layer	Up to 550 kPa	46.6 MPa^−1^ below 1 kPa	Not applicable	Wide-range capacitive sensing with low hysteresis and high cyclic stability
This work	Piezoresistive	CNT/PDMS composite sensor integrated into a soft pneumatic gripper	0–500 kPa characterization; 0–60 kPa grasping experiments	0.016 kPa^−1^	3.60 for 1 wt% CNT/PDMS	Internal-sensor-based grasp onset detection and quantitative object-size estimation without external vision

## Data Availability

The original contributions presented in this study are included in the article. Further inquiries can be directed to the corresponding authors.

## References

[1] Shintake J., Cacucciolo V., Floreano D., Shea H. (2018). Soft robotic grippers. Adv. Mater..

[2] AboZaid Y.A., Aboelrayat M.T., Fahim I.S., Radwan A.G. (2024). Soft robotic grippers: A review on technologies, materials, and applications. Sens. Actuators A Phys..

[3] Dzedzickis A., Petronienė J.J., Petkevičius S., Bučinskas V. (2024). Soft grippers in robotics: Progress of last 10 years. Machines.

[4] Zhou S., Li Y., Wang Q., Lyu Z. (2024). Integrated actuation and sensing: Toward intelligent soft robots. Cyborg Bionic Syst..

[5] Silva A., Fonseca D., Neto D.M., Babcinschi M., Neto P. (2024). Integrated design and fabrication of pneumatic soft robot actuators in a single casting step. Cyborg Bionic Syst..

[6] Liu Y., Hou J., Li C., Wang X. (2023). Intelligent soft robotic grippers for agricultural and food product handling: A brief review with a focus on design and control. Adv. Intell. Syst..

[7] Navas E., Fernández R., Sepúlveda D., Armada M., Gonzalez-de-Santos P. (2021). Soft grippers for automatic crop harvesting: A review. Sensors.

[8] Ashuri T., Armani A., Jalilzadeh Hamidi R., Reasnor T., Ahmadi S., Iqbal K. (2020). Biomedical soft robots: Current status and perspective. Biomed. Eng. Lett..

[9] Dietrich F., Müller A. (2022). Upscaling of soft material grippers to heavy duty applications in handling and assembly. CIRP Ann..

[10] Mostaghniyazdi D., Nodehi S.E. (2025). Resistive Sensing in Soft Robotic Grippers: A Comprehensive Review of Strain, Tactile, and Ionic Sensors. Electronics.

[11] Hegde C., Su J., Tan J.M.R., He K., Chen X., Magdassi S. (2023). Sensing in soft robotics. ACS Nano.

[12] Rana M.T., Islam M.S., Rahman A. (2025). Human-Centered Sensor Technologies for Soft Robotic Grippers: A Comprehensive Review. Sensors.

[13] Chen Y., Guo S., Li C., Yang H., Hao L. (2018). Size recognition and adaptive grasping using an integration of actuating and sensing soft pneumatic gripper. Robot. Auton. Syst..

[14] Matsuno T., Wang Z., Hirai S. (2017). Grasping state estimation of printable soft gripper using electro-conductive yarn. Robot. Biomim..

[15] Elgeneidy K., Neumann G., Pearson S., Jackson M., Lohse N. Contact detection and size estimation using a modular soft gripper with embedded flex sensors. Proceedings of the 2018 IEEE/RSJ International Conference on Intelligent Robots and Systems (IROS).

[16] Hosseini E.S., Manjakkal L., Shakthivel D., Dahiya R. (2020). Glycine–chitosan-based flexible biodegradable piezoelectric pressure sensor. ACS Appl. Mater. Interfaces.

[17] Yang Y., Pan H., Xie G., Jiang Y., Chen C., Su Y., Wang Y., Tai H. (2020). Flexible piezoelectric pressure sensor based on polydopamine-modified BaTiO3/PVDF composite film for human motion monitoring. Sens. Actuators A Phys..

[18] Fan F.-R., Lin L., Zhu G., Wu W., Zhang R., Wang Z.L. (2012). Transparent triboelectric nanogenerators and self-powered pressure sensors based on micropatterned plastic films. Nano Lett..

[19] Xiong X., Liang J., Wu W. (2023). Principle and recent progress of triboelectric pressure sensors for wearable applications. Nano Energy.

[20] Mishra R.B., El-Atab N., Hussain A.M., Hussain M.M. (2021). Recent progress on flexible capacitive pressure sensors: From design and materials to applications. Adv. Mater. Technol..

[21] Xia L., Xiao W., Li L., Liu X., Zhuang Q., Huang Y., Lan T., Du X., Zhao Y., Wu D. (2025). High-performance flexible capacitive pressure sensor based on a spiked nickel/polyimide composite nanofiber membrane. ACS Sens..

[22] Zhao Y., Miao L., Xiao Y., Sun P. (2024). Research progress of flexible piezoresistive pressure sensor: A review. IEEE Sens. J..

[23] Yang Y., Liu Y., Yin R. (2025). Fiber/yarn and textile-based piezoresistive pressure sensors. Adv. Fiber Mater..

[24] Chen Z., Wang Z., Li X., Lin Y., Luo N., Long M., Zhao N., Xu J.-B. (2017). Flexible piezoelectric-induced pressure sensors for static measurements based on nanowires/graphene heterostructures. ACS Nano.

[25] Liu Y., Wang J., Liu T., Wei Z., Luo B., Chi M., Zhang S., Cai C., Gao C., Zhao T. (2025). Triboelectric tactile sensor for pressure and temperature sensing in high-temperature applications. Nat. Commun..

[26] Wang H., Li Z., Liu Z., Fu J., Shan T., Yang X., Lei Q., Yang Y., Li D. (2022). Flexible capacitive pressure sensors for wearable electronics. J. Mater. Chem. C.

[27] Shi L., Li Z., Chen M., Qin Y., Jiang Y., Wu L. (2020). Quantum effect-based flexible and transparent pressure sensors with ultrahigh sensitivity and sensing density. Nat. Commun..

[28] He J., Zhang Y., Zhou R., Meng L., Chen T., Mai W., Pan C. (2020). Recent advances of wearable and flexible piezoresistivity pressure sensor devices and its future prospects. J. Mater..

[29] Chen W., Yan X. (2020). Progress in achieving high-performance piezoresistive and capacitive flexible pressure sensors: A review. J. Mater. Sci. Technol..

[30] Gao L., Zhu C., Li L., Zhang C., Liu J., Yu H.-D., Huang W. (2019). All paper-based flexible and wearable piezoresistive pressure sensor. ACS Appl. Mater. Interfaces.

[31] Chen L., Huang Y., Ning H., Liu Y., Tang H., Zhou R., Jin S., Zheng J., Yao R., Peng J. (2025). Flexible piezoresistive sensor based on cnt/pva composite with wide linear detection range for human motion monitoring. Polymers.

[32] Wang X., Lim E.G., Hoettges K., Song P. (2023). A review of carbon nanotubes, graphene and nanodiamond based strain sensor in harsh environments. C.

[33] Kang N., Zhang S., Tang F., Wang J., Li L. (2022). Silver-Hydrogel/PDMS film with high mechanical strength for anti-interference strain sensor. Colloids Surf. A Physicochem. Eng. Asp..

[34] Li L., Deng J., Kong P., Zou W., Du Z., Wang H., Zhang C. (2024). Highly sensitive porous PDMS-based piezoresistive sensors prepared by assembling CNTs in HIPE template. Compos. Sci. Technol..

[35] Yu R., Xia T., Wu B., Yuan J., Ma L., Cheng G.J., Liu F. (2020). Highly sensitive flexible piezoresistive sensor with 3D conductive network. ACS Appl. Mater. Interfaces.

[36] Zhao X., Mei D., Tang G., Zhao C., Wang J., Luo M., Li L., Wang Y. (2023). Strain and pressure sensors based on MWCNT/PDMS for human motion/perception detection. Polymers.

[37] De Rijk T.M., Lang W. (2021). Low-cost and highly sensitive pressure sensor with mold-printed multi-walled carbon nanotubes dispersed in polydimethylsiloxane. Sensors.

[38] Ma L., Yu X., Yang Y., Hu Y., Zhang X., Li H., Ouyang X., Zhu P., Sun R., Wong C.-p. (2020). Highly sensitive flexible capacitive pressure sensor with a broad linear response range and finite element analysis of micro-array electrode. J. Mater..

[39] Farman M., Surendra, Prajesh R., Upadhyay A.K., Kumar P., Thouti E. (2023). All-polydimethylsiloxane-based highly flexible and stable capacitive pressure sensors with engineered interfaces for conformable electronic skin. ACS Appl. Mater. Interfaces.

[40] Ha J.-H., Lee S.-E., Park S.-H. (2019). Effect of dispersion by three-roll milling on electrical properties and filler length of carbon nanotube composites. Materials.

[41] Wang C., Hou X., Cui M., Yu J., Fan X., Qian J., He J., Geng W., Mu J., Chou X. (2020). An ultra-sensitive and wide measuring range pressure sensor with paper-based CNT film/interdigitated structure. Sci. China Mater..

[42] Wang Z., Syed A., Bhattacharya S., Chen X., Buttner U., Iordache G., Salama K., Ganetsos T., Valamontes E., Georgas A. (2020). Ultra miniaturized InterDigitated electrodes platform for sensing applications. Microelectron. Eng..

[43] Mosadegh B., Polygerinos P., Keplinger C., Wennstedt S., Shepherd R.F., Gupta U., Shim J., Bertoldi K., Walsh C.J., Whitesides G.M. (2014). Pneumatic networks for soft robotics that actuate rapidly. Adv. Funct. Mater..

[44] Bauhofer W., Kovacs J.Z. (2009). A review and analysis of electrical percolation in carbon nanotube polymer composites. Compos. Sci. Technol..

[45] Folorunso O., Hamam Y., Sadiku R., Ray S.S., Joseph A.G. (2019). Parametric analysis of electrical conductivity of polymer-composites. Polymers.

[46] Hu N., Karube Y., Yan C., Masuda Z., Fukunaga H. (2008). Tunneling effect in a polymer/carbon nanotube nanocomposite strain sensor. Acta Mater..

[47] Bao W., Meguid S., Zhu Z., Weng G. (2012). Tunneling resistance and its effect on the electrical conductivity of carbon nanotube nanocomposites. J. Appl. Phys..

[48] Stauffer D., Aharony A. (2018). Introduction to Percolation Theory.

[49] Nan X., Zhang Y., Shen J., Liang R., Wang J., Jia L., Yang X., Yu W., Zhang Z. (2024). A review of the establishment of effective conductive pathways of conductive polymer composites and advances in electromagnetic shielding. Polymers.

[50] Oh J., Kim D.-Y., Kim H., Hur O.-N., Park S.-H. (2022). Comparative study of carbon nanotube composites as capacitive and piezoresistive pressure sensors under varying conditions. Materials.

[51] Kim K.-H., Hong S.K., Jang N.-S., Ha S.-H., Lee H.W., Kim J.-M. (2017). Wearable resistive pressure sensor based on highly flexible carbon composite conductors with irregular surface morphology. ACS Appl. Mater. Interfaces.

[52] Ramalingame R., Hu Z., Gerlach C., Rajendran D., Zubkova T., Baumann R., Kanoun O. (2019). Flexible piezoresistive sensor matrix based on a carbon nanotube PDMS composite for dynamic pressure distribution measurement. J. Sens. Sens. Syst..

[53] Jung Y., Jung K.K., Kim D.H., Kwak D.H., Ko J.S. (2020). Linearly sensitive and flexible pressure sensor based on porous carbon nanotube/polydimethylsiloxane composite structure. Polymers.

[54] Chen Y.-F., Huang M.-L., Cai J.-H., Weng Y.-X., Wang M. (2022). Piezoresistive anisotropy in conductive silicon rubber/multi-walled carbon nanotube/nickel particle composites via alignment of nickel particles. Compos. Sci. Technol..

[55] Du J., Wang L., Shi Y., Zhang F., Hu S., Liu P., Li A., Chen J. (2020). Optimized CNT-PDMS flexible composite for attachable health-care device. Sensors.

[56] Fuss F.K., Tan A.M., Weizman Y. (2019). ‘Electrical viscosity’of piezoresistive sensors: Novel signal processing method, assessment of manufacturing quality, and proposal of an industrial standard. Biosens. Bioelectron..

[57] Zhang J., Wang Z., Shang C., Qian Z., Wu Z., Yu X., Peng Z. (2024). Piezoresistive relaxation and creep model of porous polymer nanocomposite supported by experimental data. Sens. Actuators A Phys..

[58] Couri B.M., Bitzos S., Bhardwaj D., Lockhart E., Yue A., Goping I. (2018). Performance analysis of the T-DOC^®^ air-charged catheters: An alternate technology for urodynamics. Neurourol. Urodyn..

[59] Mu Q., Hu T., Tian X., Li T., Kuang X. (2023). The effect of filler dimensionality and content on resistive viscoelasticity of conductive polymer composites for soft strain sensors. Polymers.

[60] Kanoun O., Bouhamed A., Ramalingame R., Bautista-Quijano J.R., Rajendran D., Al-Hamry A. (2021). Review on conductive polymer/CNTs nanocomposites based flexible and stretchable strain and pressure sensors. Sensors.

[61] Zhu Y., Feng K., Hua C., Wang X., Hu Z., Wang H., Su H. (2022). Model analysis and experimental investigation of soft pneumatic manipulator for fruit grasping. Sensors.

[62] Lei J., Ge Z., Fan P., Zou W., Jiang T., Dong L. (2022). Design and manufacture of a flexible pneumatic soft gripper. Appl. Sci..

[63] Chicco D., Warrens M.J., Jurman G. (2021). The coefficient of determination R-squared is more informative than SMAPE, MAE, MAPE, MSE and RMSE in regression analysis evaluation. PeerJ Comput. Sci..

[64] De Myttenaere A., Golden B., Le Grand B., Rossi F. (2016). Mean absolute percentage error for regression models. Neurocomputing.

